# Spiders (Araneae) of the northeast of the Luhansk Oblast (Ukraine)

**DOI:** 10.3897/BDJ.11.e99304

**Published:** 2023-02-03

**Authors:** Nina Polchaninova, Oleksii Marushchak

**Affiliations:** 1 V.N. Karazin Kharkiv National University, Kharkiv, Ukraine V.N. Karazin Kharkiv National University Kharkiv Ukraine; 2 I. I. Schmalhauzen Institute of Zoology, NAS of Ukraine, Kyiv, Ukraine I. I. Schmalhauzen Institute of Zoology, NAS of Ukraine Kyiv Ukraine

**Keywords:** Arachnida, conservation management, diversity, protected areas, arachnofauna, steppe region

## Abstract

**Background:**

The dataset contains records of spiders collected in the northeast of Luhansk Oblast in the periods 1982-1989, 2009-2011 and 2021. It aimed at the inventory of spider fauna of the Striltsivskyi Steppe Nature Reserve and species distribution in the main grassland and forest habitats of the region. The research was also concerned with the impact of conservation management ‒ hay mowing or strict protection and man-induced steppe fire on spider communities.

**New information:**

The dataset includes records from seven geographical localities in the northeast of Luhansk Oblast with 1,955 occurrences of 6662 individuals. For the first time, it provides detailed information about spider species composition, phenology and habitat distiribution within the study area, including two conservation areas and the primary material on the studies on the impact of hay making and steppe fire on spider communities. All the records of 246 spider species with georeferencing were published in GBIF.

## Introduction

The lands of the Starobilsk Raion of the Luhansk Region, like the whole steppe zone of Ukraine, are highly transformed by human economic activity. The ploughing campaign was launched there in the 1930s and resulted in nearly all natural steppes being converted into agricultural fields. Now zonal forb-fescue-feather grass steppes on the flat interfluves are extant only in the Striltsivskyi Steppe Nature Reserve. Nevertheless, due to the rolling relief and the horse breeding developed in the late 17^th^ – early 20^th^ century, the Starobilsk Steppes are less ploughed than the southern Ukrainian steppes (Borovyk 2018). Remains of the virgin steppes, intensively grazed up to the 1990s, have been preserved on the gully slopes and bottoms. Natural arboreal and shrub vegetation forms narrow strips along the river banks and small patchy forests and shrub thickets in the gullies.

The vegetation of the Starobilsk steppes was investigated first in 1926‒1927 (Lavrenko and Dokhman 1933). Subsequently, it has been constantly monitored since the establishment of the Striltsivskyi Steppe protected area, now a department of the Luhansk Nature Reserve (Tkachenko 2009). In contrast, invertebrates were studied at irregular intervals, only some groups of beetles (Glotov 2021) and mites (Yaroshenko and Schtirts 2008) having been sampled thoroughly and analysed. Since various groups of organisms respond differently to the impact of natural and anthropogenic factors, comprehensive multitaxon studies are of critical importance for understanding changes in local biota, assessment of conservation effects (Margules and Pressey 2000, Braby and Williams 2016) and development of optimal management plans of protected areas and ecologically friendly farming (Batáry et al. 2012).

Being abundant and diverse predators, spiders play an imp­ortant role in trophic webs (Wise 1993, Mallis and Hurd 2005). They have various ecological traits and inhabit all vegetation layers in nearly all terrestrial habitats. Spiders’ diversity and ecological role make them a suitable model group for bioindication (Marc et al. 1999, Zografou et al. 2017, Cabrero-Sañudo et al. 2022).

Arachnological research in the northeast of the Luhansk Oblast was launched by the first author (NP) in the 1980s. Before that, only one locality in the southeast of the Oblast (Provllia Village, at that time Don Host Area) was investigated and a list of 55 species was published (Greze 1909). In 1982‒1989, the studies aimed at the inventory of spider fauna of the Striltsivskyi Steppe department of the Luhansk Nature Reserve and clarifying the impact of mowing management on spider assemblages (Polchaninova 1990a, Polchaninova 1995, Polchaninova 2012a). Hereinafter, E. Prokopenko (Donetsk National University, Ukraine) continued spider inventory (Prokopenko 2001) and the results were summarised in a survey of the state of knowledge of the spider fauna of Ukrainian steppe reserves (Polchaninova and Prokopenko 2007) and in the spider checklist of the Luhansk Nature Reserve (Polchaninova and Prokopenko 2011). In total, 334 spider species of 28 families were recorded from the Reserve, with 190 species registered in the Striltsivskyi Steppe.

After the devastating fire of 2008 that destroyed all vegetation in the Reserve and its vicinity, arachnological studies were focused on the effect of large-scale steppe fires on spider communities (Polchaninova 2013a, Polchaninova 2013b, Polchaninova 2015, Zografou et al. 2017). In addition, the study localities were expanded to collect spiders in other habitats typical of the region (chalk grasslands, clayey slopes, forests in the gullies, pine plantations etc.). The obtained data on spider inventory and species habitat preferences were included in the Catalogue of Spiders of Left-Bank Ukraine (Polchaninova and Prokopenko 2013, Polchaninova and Prokopenko 2017) and used for the comparison of the spider faunas of various steppe reserves (Polchaninova 1990b, Polchaninova 2012b, Prokopenko and Polchaninova 2017, Polchaninova 2021). At present, 223 spider species are known from the Striltsivskyi Steppe Reserve; other 23 species listed in the catalogue were found outside its territory. This is more than half of the species recorded from the Luhansk Oblast (402 species) (Polchaninova and Prokopenko 2019). Twelve recorded species are rare in Ukraine and 16 species are rare or patchily distributed in the study area. They can serve as bioindicators to determine areas of conservation concern.

## General description

### Purpose

The presented data will contribute to the understanding of the spider fauna of eastern Ukraine, assessment of the effectiveness of conservation management in protected areas and identification of the sites of conservation concern. Moreover, the research on spider post-fire recovery will serve as a basis for evaluating the aftermath of hostilities that arose due to Russia’s invasion of Ukraine.

## Project description

### Title

“Northern Eurasia 2022”

## Sampling methods

### Study extent

**Figure 1.**
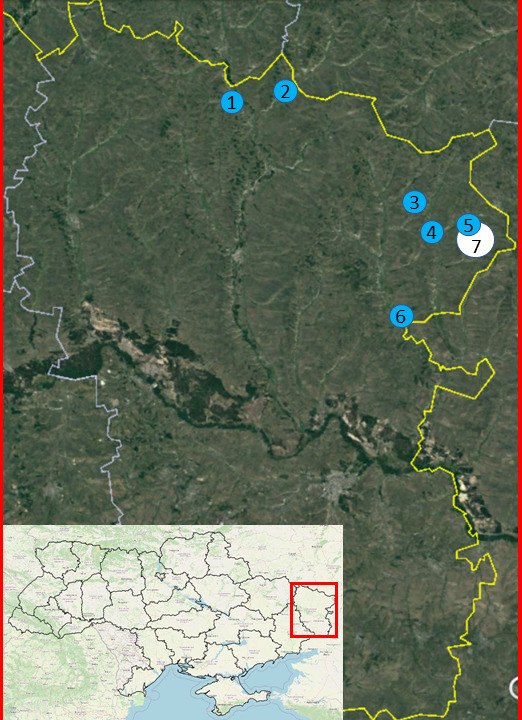
Map of collecting localities in the northeast of Luhansk Oblast: 1 - Taniushivka, 2 - Novobila, 3 - Zorykivka, 4 - Striltsivka, 5 - Velykotsk, 6 - Horodyshche, 7 - Krynychne, site of stationary monitoring the Striltsivskyi Steppe department of the Luhansk Nature Reserve and its vicinity.

The dataset is based on the records of spiders from the northern and north-eastern parts of the Luhansk Oblast of Ukraine. The study sites are located in the vicinities of the villages of Novobila, Taniushivka, Zorykivka, Striltsivka, Velykotsk, Krynychne and Horodyshche of the Starobilsk Raion (Fig. 1). A long-term stationary investigation was conducted only in the Striltsivskyi Steppe Department of the Luhansk Nature Reserve and adjacent territory (near Krynychne Village) in two periods: 1982‒1989 and 2009‒2011. The study sites near Zorykivka and Velykotsk were investigated during one field season (May‒September) in 2009 and 2011, respectively, the Kreidovi Vidslonennia Botanical Preserve of Local Importance (Striltsivka) was sampled in May‒August 2011 and September‒October 2021. Other localities were visited only once for ad-hoc collection. The dataset provides 1955 occurrences of 6662 recorded individuals (Polchaninova 2022). In total (excluding iNaturalist), there are only 5,561 occurrences of spiders from Ukraine in GBIF (Gbif.org 2022), so this dataset comprises about one-third of the information published in this format. Eleven types of habitats were investigated: forb-fescue-feather grass steppe on the flat interfluves and the gully slopes, meadow-like vegetation at the gully bottoms, chalk hills with calcareous vegetation, clayey gully slopes with xerophytic steppe vegetation, pasture on mesic floodplain meadows, abandoned fields, river banks with riparian vegetation, forest shelterbelts, pine forest plantations, bairak forests and their edges (semi-natural forests covering gully bottoms and slopes in the south of the Forest-Steppe and the north of the Steppe zone) and synanthropic habitats. The habitat types are adopted from the National Habitat Catalogue of Ukraine (Kuzemko et al. 2018). Depending on the research targets (faunal inventory or impact of disturbance factors), we arranged 32 sampling plots, the greatest attention being paid to the steppe biotopes (Table 1). In the Nature Reserve, the sampling took into account topography and conservation management of the study plot (strictly protected or mowed steppe) that is specified in Table 1. We also marked with the letter *B* the sampling localities in Striltsivskyi Steppe burnt in August 2008. In the Table of species habitat distribution (Table 2), we combined in one column shelter forest belts+forest edges as ecotone habitats and pine plantations+bairak forests as the forest ones.

### Sampling description

Spiders were collected by standard collecting methods: sweep netting, pitfall trapping, quadrat sampling and by hand. Plastic cups of 6.5 cm diameter were used as traps and 4% formalin was used for preservation. At each study plot, we set a line of 10 traps at a 10 m distance. The traps were exposed for 3‒5 days once per month in the study period 1982‒1989. In 2009‒2011, the traps were checked approximately once a month from early May to early July and from September to October. Sweep netting was conducted with a 30-cm diameter entomological net, three to five samples of 50 sweeps per plot/month. The quadrat sampling was performed by collecting spiders by hand from 25 x 25 cm plots on the ground and in the litter and thatch.The number of individuals given in the dataset can be normalised to 100 trap-days, other sampling efforts are not specified. The hand collecting was only qualitative.

### Quality control

The collected spiders were preserved in 70% ethanol and identified by the first author (NP). Taxonomy nomenclature follows the World Spider Catalog (http://wsc.nmbe.ch, accessed 10.11.2022). The material is deposited in N. Polchaninova’s private collection (Kharkiv, Ukraine).

### Step description

1. Field expeditions to the study sites.
2. Establishing a line of ten traps at a distance of 10 m at each study plot.
3. Regular checking of the traps.
4. Sweep netting conducted in the same habitats, three to five samples of 25 double sweeps per plot; hand collecting.
5. Georeferencing with the help of GPS-navigator.
6. Species identification in the laboratory.
7. Organising of a dataset according to Darwin Core standards.

## Geographic coverage

### Description

All collecting localities are limited to the Starobilsk Raion of the Luhansk Oblast. Two localities lie in the north of the region, one in the south and the others in the northeast. The area in question is located on the southern spurs of the Central Russian Upland. The climate is moderate continental. In terms of physical-geographical division, the area belongs to the Starobilsk slope-and-upland oblast of the Transdonets-Don krai, of the Northern steppe subzone of the Steppe zone (Marynych et al. 2003). In terms of geo-botanical zoning, it refers to the Siverskyi Donets okrug of the forb-bunchgrass steppes, bairak oak forests and vegetation of the chalk outcrops (tomillares) of the Middle Don subprovince of the Pontic steppe province of the Eurasian steppe region (Didukh and Shelyag-Sosonko 2003).

### Coordinates

47.806 and 50.078 Latitude; 37.837 and 40.166 Longitude.

## Taxonomic coverage

### Description

The dataset includes records of 6662 spiders belonging to 246 species of 137 genera and 27 families (Table 2). Most individuals were identified at the species level.

### Taxa included

**Table:** 

Rank	Scientific Name	
kingdom	Animalia	
phylum	Arthropoda	
class	Arachnida	
order	Araneae	
family	Araneidae	
family	Agelenidae	
family	Anyphaenidae	
family	Atypidae	
family	Cheiracanthiidae	
family	Clubionidae	
family	Dictynidae	
family	Eresidae	
family	Gnaphosidae	
family	Hahniidae	
family	Lycosidae	
family	Linyphiidae	
family	Liocranidae	
family	Mimetidae	
family	Miturgidae	
family	Oxyopidae	
family	Philodromidae	
family	Pholcidae	
family	Phrurolithidae	
family	Pisauridae	
family	Salticidae	
family	Sparassidae	
family	Tetragnathidae	
family	Theridiidae	
family	Thomisidae	
family	Titanoecidae	
family	Uloboridae	

## Temporal coverage

**Data range:** 1982-7-02 – 2021-9-24.

## Usage licence

### Usage licence

Open Data Commons Attribution License

## Data resources

### Data package title

Spiders (Araneae) of the northeast of the Luhansk Oblast (Ukraine)

### Resource link

https://www.gbif.org/uk/dataset/765d4ecb-a667-4f23-b95e-5254e7140d7e

### Alternative identifiers

https://doi.org/10.15468/8upy6t

### Number of data sets

1

### Data set 1.

#### Data set name

Spiders (Araneae) of the northeast of the Luhansk Oblast (Ukraine)

#### Data format

Darwin Core

#### Data format version

1.9

**Data set 1.** 

Column label	Column description
occurrenceID	http://rs.tdwg.org/dwc/terms/occurrenceID; an identifier of a particular occurrence, unique within this dataset. The code as made of an English variant of the author's surname, Araneae order and sequence number.
basisOfRecord	http://rs.tdwg.org/dwc/terms/basisOfRecord;the method in which data were acquired. Only "Occurrence" type was used.
scientificName	http://rs.tdwg.org/dwc/terms/scientificName;scientific names of the registered species according to the World Spider Catalogue (WSC 2022) and corrections of some spelling mistakes amd mismatches using GBIF Species Matching tool.
scientificNameAuthorship	http://rs.tdwg.org/dwc/terms/scientificNameAuthorship; the authorship information for the provided scientific name of the registered species.
acceptedNameUsage	http://rs.tdwg.org/dwc/terms/acceptedNameUsage;the full name of the currently valid taxon of registered species from Araneae order.
kingdom	http://rs.tdwg.org/dwc/terms/kingdom; the full scientific name of the kingdom in which the taxon is classified.
phylum	http://rs.tdwg.org/dwc/terms/phylum; the full scientific name of the phylum or division in which the taxon is classified.
class	http://rs.tdwg.org/dwc/terms/class;the full scientific name of the class in which the taxon is classified.
order	http://rs.tdwg.org/dwc/terms/order; the full scientific name of the order in which the taxon is classified.
family	http://rs.tdwg.org/dwc/terms/family; the full scientific name of the family in which the taxon is classified.
genus	http://rs.tdwg.org/dwc/terms/genus;the full scientific name of the genus in which the taxon is classified.
specificEpithet	http://rs.tdwg.org/dwc/terms/specificEpithet; the name of the first or species epithet of the scientificName.
taxonRank	http://rs.tdwg.org/dwc/terms/taxonRank; the taxonomic rank of the most specific name in the scientificName.
decimalLatitude	http://rs.tdwg.org/dwc/terms/decimalLatitude; geographic latitude in decimal degrees.
decimalLongitude	http://rs.tdwg.org/dwc/terms/decimalLongitude; geographic longitude in decimal degrees.
geodeticDatum	http://rs.tdwg.org/dwc/terms/geodeticDatum;spatial reference system upon which the geographic coordinates are given (WGS84).
coordinateUncertaintyInMetres	http://rs.tdwg.org/dwc/terms/coordinateUncertaintyInMeters; the horizontal distance (in metres) from the given decimalLatitude and decimalLongitude describing the smallest circle containing the whole of the location.
recordedBy	http://rs.tdwg.org/dwc/iri/recordedBy;authorship of the record.
georeferencedBy	http://rs.tdwg.org/dwc/terms/georeferencedBy;the person, who provided the records with correct coordinates.
identifiedBy	http://rs.tdwg.org/dwc/iri/identifiedBy;authorship of the taxon identification.
language	http://purl.org/dc/terms/language; the language of the resource.
verbatimEventDate	http://rs.tdwg.org/dwc/terms/verbatimEventDate;the original form or even date record in the author's notes from the fieldwork.
eventDate	http://rs.tdwg.org/dwc/terms/eventDate;the date-time when the event was recorded.
year	http://rs.tdwg.org/dwc/terms/year;the year in which the record was made.
type	http://purl.org/dc/elements/1.1/type; the nature or genre of the resource.
modified	http://purl.org/dc/terms/modified;the year on which the resource was changed.
habitat	http://rs.tdwg.org/dwc/terms/habitat;the description of the habitat, where the record was made.
continent	http://rs.tdwg.org/dwc/terms/continent;the name of the continent in which the location is situated.
country	http://rs.tdwg.org/dwc/terms/country; the name of the country in which the location is situated (Ukraine).
countryCode	http://rs.tdwg.org/dwc/terms/countryCode;the standard code for the country in which the location is situated (UA).
stateProvince	http://rs.tdwg.org/dwc/terms/stateProvince;the name of the smaller administrative region than country in which the location is situated (Luhansk Oblast).
locality	http://rs.tdwg.org/dwc/terms/locality; the specific description of the place, where the record was made.
verbatimLocality	http://rs.tdwg.org/dwc/terms/verbatimLocality; original textual description of the place according to the author's notes from the fieldwork.
samplingProtocol	http://rs.tdwg.org/dwc/iri/samplingProtocol; methods used during the research.
samplingEffort	http://rs.tdwg.org/dwc/terms/samplingEffort; amount of effort expended during the fieldwork.
occurrenceStatus	http://rs.tdwg.org/dwc/terms/occurrenceStatus; statement about the presence or absence of a taxon at a location.
disposition	http://rs.tdwg.org/dwc/terms/disposition; the current state of a specimen after the record was made.
organismQuantityType	http://rs.tdwg.org/dwc/iri/organismQuantityType; the type of quantification system used for the quantity of organisms.
organismQuantity	http://rs.tdwg.org/dwc/terms/organismQuantity; enumeration value for the quantity of organisms.
organismRemarks	http://rs.tdwg.org/dwc/terms/organismRemarks;additional notes about the recorded animals.
sex	http://rs.tdwg.org/dwc/iri/sex;the sex of the biological individual(s).
lifeStage	http://rs.tdwg.org/dwc/terms/lifeStage;the life stage of the registered animals.

## Figures and Tables

**Table 1.** Sampling plots.

Longitude	Latitude	Locality	Habitats	Sampling year
49.29750	40.07278	Krynychne	strictly protected (unmowed steppe) on the flat interfluves *B*	1982-1989, 2009, 2011
49.29722	40.07139	Krynychne	periodically mowed steppe on a gully slope; mowing was ceased in the 1990s *B*	1982-1989, 2009, 2011
49.29000	40.08722	Krynychne	periodically mowed steppe on the flat interfluves *B*	2009, 2011
49.28333	40.08061	Krynychne	periodically mowed steppe on a gully slope *B*	2009
49.29694	40.07000	Krynychne	*B* gully bottom with meadow-steppe vegetation	1982-1989, 2009, 2011
49.29278	40.08806	Krynychne	*B* gully bottom with meadow-steppe vegetation	2009, 2011
49.29611	40.06611	Krynychne	abandoned field *B*	2011
49.30222	40.06833	Krynychne	chalk slope *B*	1982-1989, 2009
49.30389	40.07722	Krynychne	pasture on the floodplain meadow and adjacent steppe slopes	1982-1989, 2009
49.30694	40.08389	Krynychne	riverbank with arboreal and herbaceous vegetation	1982-1989, 2009
49.30083	40.07028	Krynychne	forest shelterbelt	1982-1989
49.30083	40.69333	Krynychne	country house	1982-1989
49.29475	40.03912	Krynychne	edge of a bairak forest	2009, 2011
49.29422	40.04094	Krynychne	Caragana thickets on the top of a slope	2009
49.29433	40.04345	Krynychne	the slope of a steppe gully, gully bottom	2009
49.29422	40.04094	Krynychne	abandoned field	2009
49.31364	40.04066	Velykotsk	clayey slope	2011
49.31364	40.04066	Velykotsk	gully bottom	2011
49.37433	39.97010	Velykotsk	bairak forest	2009
49.38804	39.77307	Zorykivka	pine plantation	2009
49.39184	39.80012	Zorykivka	steppe on a gully slope	2009
49.38561	39.87377	Zorykovka	bairak forest and forest edge	2009
49.38914	39.81055	Zorykivka	gully bottom	2009
49.38724	39.81557	Zorykivka	chalk slope	2009
49.28806	39.85083	Striltsivka	chalk slope and gully bottom	2009, 2021
49.04018	39.63299	Horodyshche	steppe on a gully slope	1989
49.05027	39.65027	Horodyshche	forest plantation	1989
49.04916	39.65527	Horodyshche	riverbank	1989
49.05666	39.64750	Horodyshche	chalk slope	1989
49.76270	38.9309	Taniushivka	bairak forest	2009
49.76270	38.93090	Taniushivka	steppe gully	2009
49.76861	39.18805	Novobila	steppe on a slope, mesic meadow	2009

**Table 2.** The list of spider species, their habitat distribution and the number of collected individuals in the northeast of the Luhansk Oblast: 1 ‒ forb-fescue-feather grass steppe on the flat interfluves and gully slopes (including abandoned fields on various stages of vegetation succession); 2 – meadow steppe in the gully bottoms; 3 – chalk slopes; 4 – clayey slopes; 5 – pasture in the floodplain meadow; 6 – forest edges and shelterbelts; 7 – bairak forest and forest plantation; 8 – riverbank; 9 – synanthropic habitats.

**Families/Species**	**Types of habitats**								
	1	2	3	4	5	6	7	8	9
**Fam. Agelenidae**									
*Agelenalabyrinthica* (Clerck, 1757)	2					4		1	
*Agelenopsispotteri* (Blackwall, 1846)								3	
*Allagelenagracilens* (C. L. Koch, 1841)							1		
*Eratigenaagrestis* (Walckenaer, 1802)					1				
**Fam. Anyphaenidae**									
*Anyphaenaaccentuata* (Walckenaer, 1802)						6	1	4	
**Fam. Araneidae**									
*Agalenatearedii* (Scopoli, 1763)	28	15	9	12	3	4	1		
*Araneusalsine* (Walckenaer, 1802)			2				2		
*Araneusdiadematus* Сlerck, 1757		2	1			13	27		
*Araneusquadratus* Clerck, 1757	3	10			3	2	5		
*Araniellacucurbitina* (Clerck, 1757)	3	2				5			
*Aranielladisplicata* (Hentz, 1847)	2						1		
*Argiopebruennichi* (Scopoli, 1772)	34	23		11	12	14		1	
*Cercidiaprominens* (Westring, 1851)	7	7				2	3		
*Cyclosaconica* (Pallas, 1772)							9		
*Cyclosaoculata* (Walckenaer, 1802)	4			2	4				
*Gibbaraneabituberculata* (Walckenaer, 1802)		2		1		14	7		
*Gibbaraneaullrichi* (Hahn, 1835)					1				
*Hypsosingasanguinea* (C. L. Koch, 1844)	4								
*Hypsosingaalbovittata* (Westring, 1851)		7			1				
*Hypsosingapygmaea* (Sundevall, 1831)							1		
*Larinioidessuspicax* (O. Pickard-Cambridge, 1876)								4	
*Mangoraacalypha* (Walckenaer, 1802)	67	39	4	12	10	21	86	9	
*Neosconaadianta* (Walckenaer, 1802)	38	9	2	9	3	1			
*Singahamata* (Clerck, 1757)	19	19				2			
*Singanitidula* C. L. Koch, 1844								2	
*Zilladiodia* (Walckenaer, 1802)						1	2		
**Fam. Atypidae**									
*Atypusmuralis* Bertkau, 1890	4	12							
*Atypuspiceus* (Sulzer, 1776)						2	2		
**Fam. Cheiracanthiidae**									
*Chеiracanthium elegans* Thorell, 1875						4			
*Cheiracanthiumerraticum* (Walckenaer, 1802)	2	1							
*Cheiracanthiumpennyi* O. Pickard-Cambridge, 1873	30	9		1	5		1		
*Cheiracanthiumpunctorium* (Villers, 1789)	6	9		3				6	
*Cheiracanthiumvirescens* (Sundevall, 1833)	1	1			3		2		
**Fam. Clubionidae**									
*Clubionacaerulescens* L.Koch, 1867		1				1	2		
*Clubionafrutetorum* L. Koch, 1867	1								
*Clubionalutescens* Westring, 1851		1						1	
*Clubionaneglecta* O. Pickard-Cambridge, 1862	1	3							
*Clubionapallidula* (Clerck, 1757)							1		
*Clubionapseudoneglecta* Wunderlich, 1994	3	1							
*Clubionastagnatilis* Kulczyński, 1897								1	
*Clubionasubtilis* L. Koch, 1867	1	2							
**Fam. Dictynidae**									
Brigittea latens (Fabricus, 1775)	40	4		6	4				
*Dictynaarundinacea* (Linnaeus, 1758)	58	35	3	8	14	8	3		
*Dictynauncinata* Thorell, 1856						2	8		
*Lathyshumilis* (Blackwall, 1855)							1		
**Fam. Eresidae**									
*Eresus* sp.				1					
**Fam. Gnaphosidae**									
*Berlandinacinerea* (Menge, 1872)	3		7	3	1	1			
*Callilepisnocturna* (Linnaeus, 1758)		1				4			
*Civizelotespygmaeus* (Miller, 1943)					1				
*Drassodespubescens* (Thorell, 1856)	16	15	2				3		
*Drassylluslutetianus* (L. Koch, 1866)						2			
*Drassylluspraeficus* (L. Koch, 1866)	11	18	1			4			
*Drassylluspumilus* (C.L. Koch, 1839)						1			
*Drassylluspusillus* (C. L. Koch, 1833)	1	13	2		1	5	10		
*Drassyllusvillicus* (Thorell, 1875)						1	3		
*Drassyllusvinealis* (Kulczyn'ski, 1897)				3					
*Gnaphosadolosa* O. Herman, 1879					2	1			
*Gnaphosaleporina* (L. Koch, 1866)	159	64	22	1	4	13	10		
*Gnaphosalicenti* Schenkel, 1953	5				12				
*Gnaphosalugubris* (C. L. Koch, 1839)	7	3					1		
*Gnaphosasteppica* Ovtscharenko, Platnick & Song, 1992				49	1				
*Gnaphosataurica* Thorell, 1875	2	8		31	1	4			
*Haplodrassusdalmatensis* (L. Koch, 1866)				1					
*Haplodrassuskulczynskii* Lohmander, 1942	31	7	1	3	2	1			
*Haplodrassusminor* (O. Pickard-Cambridge, 1879)						1			
*Haplodrassussignifer* (C. L. Koch, 1839)	79	74	2			9	2		
*Haplodrassussilvestris* (Blackwall, 1833)						1	2		
*Haplodrassusumbratilis* (L. Koch, 1866)	39	93		1	1	32	11		
*Marinarozelotesmalckini* (Platnick & Murphy, 1984)		1		1	21	1			
*Micariabosmansi* Kovblyuk & Nadolny, 2008		1			1				
*Micariadives* (Lucas, 1846)	1								
*Micariaformicaria* (Sundevall, 1831)	5	1				2			
*Micariafulgens* (Walckenaer, 1802)	4	1				4	3		
*Micariapulicaria* (Sundevall, 1831)		6			1	2	2	1	
*Micariarossica* Thorell, 1875					1				
*Phaeocedusbraccatus* (L. Koch, 1866)	1						2		
*Poecilochroavariana* (C. L. Koch, 1839)	3					3			
*Zeloteselectus* (C. L. Koch, 1839)	165	69	3		1	9	1		
*Zelotesfuscus* (Thorell, 1875)	2	4				4	17		
*Zeloteslatreillei* (Simon, 1878)	8	57					1		
*Zeloteslongipes* (L. Koch, 1866)	12	9		2	2	1			
*Zelotespetrensis* (C. L. Koch, 1839)							3	1	
*Zelotespseudogallicus* Ponomarev, 2007	11	7							
*Zelotessegrex* (Simon, 1878)				1					
**Fam. Hahniidae**									
*Hahniaononidum* Simon, 1875							13		
*Hahniapusilla* C. L. Koch, 1841							1		
**Fam. Linyphiidae**									
*Abacoproecessaltuum* (L. Koch,1872)						2	4		
*Agynetafuscipalpa* (C. L. Koch, 1836)							2		
*Agynetarurestris* (C. L. Koch, 1836)	11	2			2	1	4		
*Agynetasimplicitarsis* (Simon, 1884)	3	1							
*Agynetasubtilis* (O. Pickard-Cambridge, 1863)							1		
*Centromerussylvaticus* (Blackwall, 1841)							3	1	
*Ceratinellabrevis* (Wieder, 1834)							1		
*Dactylopisthesmirificus* (Georgescu, 1976)	12						3		
*Diplocephaluspicinus* (Blackwall, 1841)							1		
*Diplostylaconcolor* (Wider, 1834)							2	1	
*Entelecaraacuminata* (Wider, 1834)							5		
*Floroniabucculenta* (Clerck, 1757)							1	1	
*Gnathonariumdentatum* (Wider, 1834)							2	1	
*Gonatiumparadoxum* (L. Koch, 1869)							3		
*Gongylidiumrufipes* (Linnaeus, 1758)							1	2	
*Helophorainsignis* (Blackwall, 1841)							8		
*Linyphiahortensis* Sundevall, 1830							13		
*Linyphiatenuipalpis* Simon, 1884	4	5			4	2	11		
*Linyphiatriangularis* (Clerck, 1757)	10	18			6		34	11	
*Masosundevalli* (Westring, 1851)							7		
*Megalepthyphantespseudocollinus* Saaristo, 1997	1								
*Metopobactrusprominulus* (O. Pickard-Cambridge, 1873							1		
*Microlinyphiapusilla* (Sundevall, 1830)	1	5					2		
*Micronetaviaria* (Blackwall, 1841)						1	14		
*Miniciacaspiana* Tanasevitch, 1990	3	1							
*Nerieneclathrata* (Sundevall, 1830)		1					5	2	
*Nerieneradiata* (Walckenaer, 1841)							9		
*Oedothoraxapicatus* (Blackwall, 1850)		1							
*Pelecopsisparallela* (Wider, 1834)							3		
*Pocadicnemispumila* (Blackwall, 1841)	1	2						6	
*Porrhomma* sp.		2							
*Stemonyphanteslineatus* (Linnaeus, 1758)	8	2	1		1	4	1		
*Tenuiphantesflavipes* (Blackwall, 1854)							5		
*Trichoncoidespiscator* (Simon, 1884)	1								
*Trichoncusvasconicus* Denis, 1944	4								
*Uralophantesponticus* Gnelitsa, 2022				1					
*Walckenaeriaantica* (Wider, 1834)							1	2	
**Fam. Liocranidae**									
*Agroecacuprea* Menge, 1873		3			1	2	2		
*Agroecalusatica* (L. Koch, 1875)						2			
*Agroecamaculata* L. Koch, 1879	23	5							
**Fam. Lycosidae**									
*Alopecosacuneata* (Clerck, 1757)	197	121	65	3		44	17		
*Alopecosacursor* (Hahn, 1831)	29	3		8	10				
*Alopecosafarinosa* (Herman, 1879)		2							
*Alopecosapulverulenta* (Clerck, 1757)	93	170	2	1	1	39	41	1	
*Alopecosaschmidti* (Hahn, 1835)				4					
*Alopecosasolitaria* (Herman, 1879)	20	3		1	3				
*Alopecosasulzeri* (Pavesi,1873)	16	76				6	11		
*Alopecosataeniopus* (Kulczyński, 1895)	33	19	1		3	4			
*Alopecosatrabalis* (Clerck, 1757)	1					30	11		
*Arctosalutetiana* (Simon, 1876)						52	101		
*Lycosasingoriensis* (Laxmann, 1770)	1								
*Pardosaagrestis* (Westring, 1861)	3	3						1	
*Pardosaamentata* (Clerck, 1757)								2	
*Pardosaitalica* Tongiorgi, 1966					3				
*Pardosalugubris* (Walckenaer, 1802)	5	24				38	159		
*Pardosapaludicola* (Clerck, 1757)		2							
*Pardosapalustris* (Linnaeus, 1758)		1					5	1	
*Pardosaprativaga* (L. Koch, 1870)		7						10	
*Piratulahygrophila* (Thorell, 1872)								1	
*Trochosarobusta* (Simon, 1876)	69	36	1	7	4	27	2		
*Trochosaruricola* (De Geer, 1778)	2					1	1		
*Trochosaterricola* Thorell, 1856	79	162	6			36	46		
*Xerolycosaminiata* (C. L. Koch, 1834)	150	48			13	1			
**Fam. Mimetidae**									
*Eroaphana* (Walckenaer, 1802)						3			
*Erofurcata* (Villers, 1789)							1		
**Fam. Miturgidae**									
*Zoraarmillata* Simon, 1878	1	1							
*Zoranemoralis* (Blackwall, 1861)							1		
*Zorapardalis* Simon, 1878	4	3	2		1	4	1		
*Zorasilvestris* Kulczyński, 1897							7		
*Zoraspinimana* (Sundevall, 1833)						1	6		
**Fam. Oxyopidae**									
*Oxyopesheterophthalmus* (Latreille, 1804)	3				6	3			
**Fam. Philodromidae**									
*Philodromusaureolus* (Clerck, 1757)							2		
*Philodromuscespitum* (Walckenaer, 1802)	32	9		1		5	23		
*Philodromusdispar* Walckenaer, 1826							12		
*Philodromusemarginatus* (Schrank, 1803)							1		
*Rhysodromushistrio* (Latreille, 1819)	6								
*Thanatusarenarius* L. Koch, 1872	187	30	6	1	3	1	3		
*Thanatussabulosus* (Menge, 1875)							5		
*Tibellusmacellus* Simon, 1875	19			3	4	5		1	
*Tibellusmaritimus* (Menge, 1875)				7				1	
*Tibellusoblongus* (Walckenaer, 1802)	29	23			3	3	7		
**Fam. Pholcidae**									
*Pholcusponticus* Thorell, 1875									2
**Fam. Phrurolithidae**									
*Phrurolithusfestivus* (C. L. Koch, 1835)		1					5		
*Phrurolithuspullatus* Kulczynski, 1897	3				3				
**Fam. Pisauridae**									
*Pisauranovicia* (L. Koch, 1878)	1	3		1		4	1		
**Fam. Salticidae**									
*Aelurilluslaniger* Logunov & Marusik, 2000	6					1	1		
*Aelurillusv-insignitus* (Clerck, 1757)	19	2		5	2				
*Asianellusfestivus* (C. L. Koch, 1834)	1	2			2				
*Attulusdzieduszykii* (L. Koch, 1870)					2				
*Attulusfloricola* (C. L. Koch, 1837)								3	
*Balluschalybeius* (Walckenaer, 1802)							3		
*Carrhotusxanthogramma* (Latreille, 1819)	1	1				1	1		
*Chalcoscirtusnigritus* (Thorell, 1875)	1								
*Euophrysfrontalis* (Walckenaer, 1802)	6	1			2				
*Evarchaarcuata* (Clerck, 1757)	8	5				5	2	6	
*Evarchafalcata* (Clerck, 1757)	4	4				2	5	4	
*Evarchalaetabunda* (C. L. Koch, 1846)	1	1							
*Evarchamichailovi* Logunov, 1992	18	3	1	2		2			
*Heliophanusauratus* C. L. Koch, 1835	3					2	7		
*Heliophanuscupreus* (Walckenaer, 1802)	5	11				13	9		
*Heliophanusflavipes* (Hahn, 1832)	26	15		5	4	1	3		
*Marpissamuscosa* (Clerck, 1757)							1	1	
*Myrmarachneformicaria* (De Geer, 1778)		1		1					
*Neonrayi* (Simon, 1875)		3					1		
*Pellenesnigrociliatus* (Simon, 1875)					1				
*Philaeuschrysops* (Poda, 1761)	21	3		4	8	5			
*Phlegrafasciata* (Нahn, 1826)	14	5			2				
*Pseudiciusencarpatus* (Walckenaer, 1802)		1					2		
*Salticusscenicus* (Clerck, 1757)									1
*Sibianoraurocinctus* (Ohlert, 1865)		2							
*Synageleshilarulus* (C. L. Koch, 1846)	2	5							
*Synagelessubcingulatus* (Simon, 1878)	4	2							
*Talaveraaequipes* (O. Pickard-Cambridge, 1871)	3	2							
**Fam. Sparassidae**									
*Micrommatavirescens* (Clerck, 1757)	1	4					3		
**Fam. Tetragnathidae**									
*Metellinasegmentata* (Clerck, 1757)							20	4	
*Pachygnathaclercki* Sundevall, 1823								1	
*Pachygnathadegeeri* Sundevall, 1830		3				1			
*Tetragnathaextensa* (Linnaeus, 1758)							7	4	
*Tetragnathamontana* Simon, 1874							5	7	
**Fam. Theridiidae**									
*Asagenaphalerata* (Panzer, 1801)	1	2		1		1			
*Crustulinaguttata* (Wider, 1834)	4	5			1	1	2	1	
*Enoplognathaovata* (Clerck, 1757)		1				4	26	6	
*Enoplognathathoracica* (Hahn, 1833)	1								
*Euryopisquinqueguttata* Thorell, 1875	2				1				
*Euryopissaukea* Levi, 1951	2				1				
*Heterotheridionnigrovariegatum* (Simon, 1873)	6	2				1	1		
*Lasaeolatristis* (Hahn, 1833)							1		
*Neottiurabimaculata* (Linnaeus, 1767)	6	3		1		1			
*Neottiurasuaveolens* (Simon, 1880)	11	4							
*Phyllonetaimpressa* (L. Koch, 1881)	20			2	12		2		
*Platnickinatincta* (Walckenaer, 1802)						2	1		
*Robertusheydemanni* Wiehle, 1965		1							
*Simitidionsimile* (C. L. Koch, 1836)	18	6		2	3	8			
*Steatodaalbomaculata* (De Geer, 1778)	2								
*Steatodacastanea* (Clerck, 1757)									3
*Theridioninnocuum* Thorell, 1875		3							
*Theridionpinastri* L. Koch, 1872							1		
*Theridionvarians* Hahn, 1833							2	1	
**Fam. Thomisidae**									
*Ebrechtellatricuspidata* (Fabricius, 1775)	1	3				1	10	2	
*Heriaeusoblongus* Simon, 1918	14				5				
*Misumenavatia* (Clerck, 1757)	4	5		2		4			
*Ozyptilaatomaria* (Panzer, 1801)	1	2							
*Ozyptilapraticola* (C. L. Koch, 1837)		1					6		
*Ozyptilascabricula* (Westring, 1851)	33	6		3	1	1	1		
*Ozyptilatuberosa* (Thorell, 1875)					1				
*Pistiustruncatus* (Pallas, 1772)						1			
*Psammitisninnii* (Thorell, 1872)	1				1				
*Spiracmemongolicus* Schenkel, 1963					1				
*Spiracmestriatipes* L. Koch, 1870	52	27		3	17	2			
*Thomisusonustus* Walckenaer, 1805	29	9		5	14	1			
*Tmaruspiger* (Walckenaer, 1802)	5	1					4		
*Xysticuscristatus* (Clerck, 1757)	51	9	4		12	1	3		
Xysticus kochi Thorell, 1872	12	5		1	4	1	1		
*Xysticuslaetus* Thorell, 1875	4				1				
*Xysticuslanio* C. L. Koch, 1835		1							
*Xysticusluctator* L. Koch, 1870							10		
*Xysticusmarmoratus* Thorell, 1875					2				
*Xysticusulmi* (Hahn, 1831)	1							1	
**Fam. Titanoecidae**									
*Titanoecaschineri* L. Koch, 1872							1		
**Fam. Uloboridae**									
*Uloboruswalckenaerius* Latreille, 1806	4			1	10				
